# Spontaneous Reduction of Inverted Uterus

**Published:** 1877-10

**Authors:** 


SPONTANEOUS REDUCTION OF INVERTED UTERUS.—Was called to attend Mrs. S. in her third labor. The first confinement had taken place about six years previously, and had been attended with some complication, which I subsequently learned was complete inversion, and which was reduced readily by the attending physician. Was called to her in her second labor, to deliver a retained placenta; no inversion: This (her third) labor was short and easy. Five minutes after, however, "flooding" commenced; and being driven to the necessity of removing speedily the placenta, found a pyriform tumor projecting through the dilated os into the vagina, with a considerable portion of the placenta adhering closely to one side of its surface. Removing the attachment by careful manipulation, I immediately plugged up the unoccupied portion with a wad of linen cloth, so imminent was the danger from exhaustion. Tampon removed in an hour's time without further hemorrhage. No attempt at reduction was deemed prudent till next morning, both on account of the extreme prostration of the patient, and the lack of medical assistance. The next morning the tumor still remained unchanged; patient having rallied under appropriate treatment. Prolonged attempts at immediate reduction were made by Dr. Lartigue and myself. so constricted were the muscles of the neck and the body so contracted, that they proved abortive, and were abandoned, with the intention of shortly renewing them. In the interim, I ordered White's uterine repositor.

The instrument not arriving for several days, the delay in making another trial was longer than was desired. The patient in the mean time progressed favorably; but slight febrile symptoms, no tenderness over uterine region; in short, expressed herself as doing as well as could be expected, or as well as at any previous confinement; and, to my disappointment, obstinately refused any further interference, not even allowing the modest request of a vaginal examination, assuring me, however, if she could not do without assistance I should be informed of the fact! About the middle of May—two months afterwards—she consented to a vaginal examination, which revealed a complete restoration of the uterus to its original position.—*Obstetrical Journal.*

